# Category and Perceptual Learning in Subjects with Treated Wilson's Disease

**DOI:** 10.1371/journal.pone.0009635

**Published:** 2010-03-10

**Authors:** Pengjing Xu, Zhong-Lin Lu, Xiaoping Wang, Barbara Dosher, Jiangning Zhou, Daren Zhang, Yifeng Zhou

**Affiliations:** 1 Department of Neurobiology and Biophysics, School of Life Sciences, University of Science and Technology of China, Hefei, Anhui, People's Republic of China; 2 Laboratory of Brain Processes (LOBES), Departments of Psychology and Biomedical Engineering, and Neuroscience Graduate Program, University of Southern California, Los Angeles, California, United States of America; 3 Department of Cognitive Science, University of California Irvine, Irvine, California, United States of America; 4 Visual Information Processing Laboratory, Institute of Biophysics, Chinese Academy of Sciences, Beijing, People's Republic of China; University of Sydney, Australia

## Abstract

To explore the relationship between category and perceptual learning, we examined both category and perceptual learning in patients with treated Wilson's disease (WD), whose basal ganglia, known to be important in category learning, were damaged by the disease. We measured their learning rate and accuracy in rule-based and information-integration category learning, and magnitudes of perceptual learning in a wide range of external noise conditions, and compared the results with those of normal controls. The WD subjects exhibited deficits in both forms of category learning and in perceptual learning in high external noise. However, their perceptual learning in low external noise was relatively spared. There was no significant correlation between the two forms of category learning, nor between perceptual learning in low external noise and either form of category learning. Perceptual learning in high external noise was, however, significantly correlated with information-integration but not with rule-based category learning. The results suggest that there may be a strong link between information-integration category learning and perceptual learning in high external noise. Damage to brain structures that are important for information-integration category learning may lead to poor perceptual learning in high external noise, yet spare perceptual learning in low external noise. Perceptual learning in high and low external noise conditions may involve separate neural substrates.

## Introduction

In category learning, observers improve their performance in classifying novel stimuli into discrete categories through trial-and-error with feedback [1], [2], [3], [4]. In perceptual learning, observers improve their discrimination or detection performance in perceptual tasks through repeated practice or training [5], [6], [7], [8], [9], [10], [11], [12]. Vital for the survival and evolution of the living organisms, both forms of learning reflect long-term changes of the adult central nervous system and have been under extensive investigation [13], [14], [15], [16].

Converging evidence from cognitive psychology, neuropsychology, and brain imaging suggests that category learning may be mediated by two separate brain systems [16], [17], [18], [19], [20], [21], [22]: Rule-based category learning is mediated by frontal brain areas such as the anterior cingulate, prefrontal cortex (PFC), and by the head of the caudate nucleus in the basal ganglia. Information-integration category learning is mediated by the tail of the caudate nucleus in the basal ganglia and a dopamine-mediated reward signal. Existing evidence also suggests that the declarative memory systems and especially working memory play major roles in rule-based category learning, whereas the non-declarative memory systems and especially procedural memory are heavily involved in information-integration category learning [23], [24].

In visual perceptual tasks, behavioral analysis suggests that perceptual learning improves performance via two separable mechanisms: tuning of the task relevant perceptual template in high external noise environments and enhancing the stimulus in zero and low external noise environments [25], [26], [27]. The neural basis for perceptual learning is less clear. Plasticity of early visual areas has generally been implicated to explain perceptual learning that is specific to retinal location or orientation [28]. However, the various specificity results are equally consistent with task-specific reweighting of the “read-out” connections from early visual areas, with no changes in the areas [29], [30]. In addition, observed changes in early visual areas following extensive practice have been too modest to account for the corresponding behavioral improvements [14], [31], [32], [33]. A recent study [34] implicated a role of sensory-motor areas in perceptual learning.

Category and perceptual learning share many similarities. In both types of learning, observers are required to perform classification of perceptual stimuli, often with feedback and/or reward. In perceptual learning, the perceptual categories are often relatively simple and clearly defined in the beginning of training, although the subtle information in the perceptual stimulus must still be learned. In category learning, the categorical structure is often more complex and not explicitly provided, but has to be discovered through practice. On the other hand, although the perceptual categories are defined in perceptual learning, observers can improve their performance via re-tuning of the perceptual template in noisy environments. The re-tuning mechanism of perceptual learning might be related to refinement of perceptual categories in category learning.

Despite the similarities of category and perceptual learning, to our knowledge, there has been no investigation on the relationship between the two forms of learning. In this study, we explored the relationship between the two forms of category learning and the two mechanisms of perceptual learning. Specifically, we examined both category and perceptual learning in patients with treated Wilson's disease (WD), whose basal ganglia, known to be important in category learning, were damaged by the disease. We measured their learning rate and accuracy in both rule-based category learning and information-integration category learning, and magnitudes of perceptual learning in a wide range of external noise conditions, and compared the results with that of normal controls. In light of the recent advances in the neuropsychological theory of category learning [16], we expect that the relationship between category learning and perceptual learning might lead to new hypotheses about the neural substrate of perceptual learning, and the learning process itself.

Wilson's disease [35], hepatolenticular degeneration, is an autosomal, recessively inherited disorder of copper metabolism with a prevalence of about 10 to 40 per million [36]. The abnormal gene that causes Wilson's disease is located on chromosome 13 band q14.3 [37], which is known to code for a copper-transporting P-type ATPase. A mutation in the WD gene (ATP7B) results in reduced excretion of copper into the bile and leads to its accumulation in the liver, kidney, cornea, bones and brain. Consequently, the clinical expression is highly variable with predominant hepatic, neurological, or psychiatric symptoms. Different parts of the central nervous system, including the cerebellum, brainstem, thalamus, and subcortical white matter, can be affected, but the greatest damage usually occurs in the basal ganglia [38], [39], [40], [41]. Recent research revealed that patients with basal ganglia pathology show significant cognitive impairments, mostly in language, motor and memory functions [42], [43], [44].

The nuclei of the basal ganglia, the head and tails of the caudate nucleus, are known to be important in both rule-based and information-integration category learning [45], [46]. We therefore expected that subjects with treated Wilson's disease may have deficits in both rule-based and information-integration category learning, although the deficits may not be correlated because the impact of the disease on different nuclei of the basal ganglia depends on the exact nature of the damage in each individual patient. We evaluated their performance in both category learning and perceptual learning, and the relationship between different types of category and perceptual learning.

## Methods

### Observers

**Figure 1:**
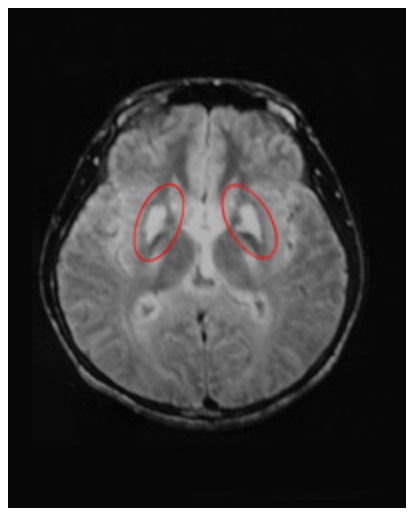
An MRI image of a typical WD subject. The image shows bilateral basal ganglion damage.

Thirteen male and seven female symptomatic Wilson's Disease patients (Median age = 21.7 yrs, SD = 6.9 yrs, Range: 14 to 46 yrs) were recruited from the Institute of Neurology, University Hospital, Anhui College of Traditional Chinese Medicine (Hefei, Anhui, China). Informed consent was obtained for participation in the study. The diagnoses of the patients were based on the presence of classical copper-related biochemical indicators (plasmaceruloplasma<200 mg/l and/or serum copper oxidase<0.20 OD, 24 hour urine copper excretion ≥100µg (1.56µmol), and/or liver copper concentrations>250 ug/g on needle biopsy) and the presence of Kayser-Fleischer rings around the iris. All the patients were treated with D-Penicillamine before and during the study period. MRI or CT scans were obtained for all the patients (Figure 1). Of the 20 patients, one patient showed no significant pathology in her MRI images. The other 19 had visible lesions in basal ganglia, with some showing additional subcortical pathology in the thalamus or brainstem, and one showing cortical pathology especially in the frontal lobe in addition to subcortical lesions. The patients were also evaluated with the Clinical Dementia Rating (CDR): seven had CDR of 0, nine had CDR of 0.5, and four had CDR of 1.0. All 20 patients participated in the category learning experiments. Five male and three female patients (Median age = 20.8 yrs, SD = 5.6 yrs; Range: 14 to 27 yrs) participated in the eight-day perceptual learning experiment after finishing the category learning tasks. Among these patients, seven had only basal ganglia lesions and one had additional cortical pathology.

The control group consisted of 24 male and 14 female healthy volunteers (Median age = 24.1 yrs; SD = 2.8 yrs; Range: 17 to 40 yrs). They were closely matched to the patients in terms of gender and age. All the 38 observers in the control group finished the category learning tasks. Seven male and five female control observers (Median age = 22.0 yrs; SD = 3.2 yrs; Range: 17 to 28 yrs) also completed the perceptual learning experiment. Because the age of the groups in all the comparisons was well matched, age was not a factor in our study.

All the observers had normal or corrected-to-normal vision and were naive to the purpose of the study.

### Apparatus

All the experiments were programmed using Matlab 6.1 with Psychtoolbox extensions [47], and run on a P4 2.4G computer with a Sony G220 monitor. The background luminance of the monitor was set at 27 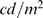. A special circuit was used to increase the graylevel resolution of the display system (>12.5 bits); A psychophysical procedure was used to linearize the monitor response function [48]. All displays were viewed binocularly with natural pupil in a dimly lighted room.

### Category Learning Tasks

The rule-based and information-integration category learning tasks were generated according to the guidelines described in [17]. Some have referred these two tasks as explicit and implicit category learning tasks [16]. Each stimulus consisted of a small geometric shape superimposed on the center of a larger geometric shape, appearing in the center of the computer display. In each trial, the observer was instructed to classify the stimulus on the screen into one of two categories. Observers were encouraged to randomly guess their responses in the beginning of each task, and were told to try to use the auditory feedback to improve their performance.

**Figure 2:**
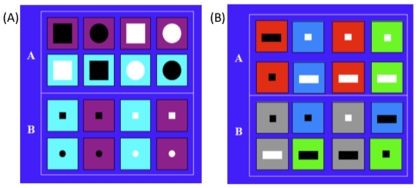
Stimuli for the category learning tasks. In the rule-based task (A), only one feature is relevant for the sorting rule (inside size). In the information-integration task (B), a combination of the two relevant features is required to classify these stimuli.

For the rule-based category learning task, four binary dimensions were used to generate the stimuli: background color (turquoise or purple), inside color (black or white), inside shape (square or circle), and inside size (big or small). There were therefore a total of 16 exemplars. The stimuli were divided into two categories based on a single dimension: the inside size; the other three dimensions were irrelevant (Figure 2A).

Two binary features and one quaternary feature were used to generate stimuli for the information-integration category learning task: inside shape (square or rectangle), inside color (black or white), and background color (red, blue, green, gray). The two categories were defined by a combination of the inside and background colors (Figure 2B). The inside shape was irrelevant. Category one consisted of all stimuli with (1) a red background regardless of all the other features, (2) a white inside and a blue background, and (3) a white inside and a green background. Category two consisted of all stimuli with (1) a gray background regardless of the other features, (2) a black inside and a blue background, and (3) a black inside and a green background.

Two scores were obtained in each task. The first was the number of trials it took for the observer to reach a criterion performance level – attaining cumulatively six blocks of trials with at least 8 out of 10 correct responses. This criterion was similar to that used in another study [49] and was usually achieved well before a participant reached the maximum of 200 trials. It served as an impartial metric that allowed us to analyze the data across observers. The second score was the average accuracy across all the trials leading to criterion. An observer ran a maximum of 200 trials in each task if she/he failed to reach the criterion performance level. The order of the two category learning tasks was counter-balanced across observers in each group.

### Perceptual Learning

**Figure 3:**
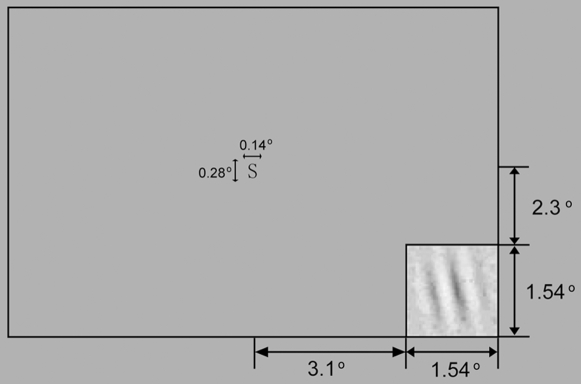
Layout of the display in the perceptual learning experiment. Subjects were asked to determine whether an S or a 5 appeared at fixation and identify the orientation of the test Gabor in the lower-right quadrant. Eight levels of external noise (“TV Snow”) were superimposed on the signal.

The perceptual learning task was identical to that of [25]. Observers discriminated the orientation of a peripheral Gabor patch embedded in visual noise while performing a central task (Fig. 3). Following a subject keypress, a fixation display (a small central square) appeared for 0.5 sec. Frames for the central task and the peripheral perceptual task appeared during the same time interval. The central task display consisted of a sequence of 3 letters and numbers with the middle letter either an S or a 5 (0.14°
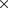
0.28°) appearing at the same location as the fixation point. The perceptual task appeared in the lower right quadrant of the monitor, and consisted of two frames of external noise, a signal frame with a Gabor patch tilted either left or right, and two additional frames of external noise. The signal was a Gabor stimulus (center frequency = 2.3 c/deg, 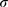
0.39°.) tilted either 12 degrees top to the left or right. All noise samples in each trial were independent samples with the same contrast (variance). The external noise was combined with the signal through temporal integration. Each frame appeared for 16.7 ms. After the stimulus sequence, the subject was cued for two responses: the central task (S vs 5) and the peripheral perceptual task (Left vs Right). Perceptual learning of orientation discrimination was measured in the lower right quadrant of the visual display. Auditory feedback for both tasks followed every trial.

On each trial, random external pixel noise was chosen from a Gaussian distribution, with one of eight levels of external noise contrast with standard deviations 0, 0.02, 0.04, 0.08, 0.12, 0.16, 0.25, or 0.33. A 3-down-1-up staircase procedure that decreased the signal contrast by 10% (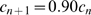) after three successive correct responses and increased the contrast by 10% (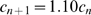) after every incorrect response was used to track the threshold at 79.3% correct in each of the eight external noise conditions.

The experiment consisted of eight training sessions, run on separate days. Each session consisted of eight interleaved external noise conditions and 100 trials per external noise condition and lasted about 40 minutes. Observers ran the perceptual learning experiment after they finished the category learning tasks.

## Results

### Category Learning

**Figure 4:**
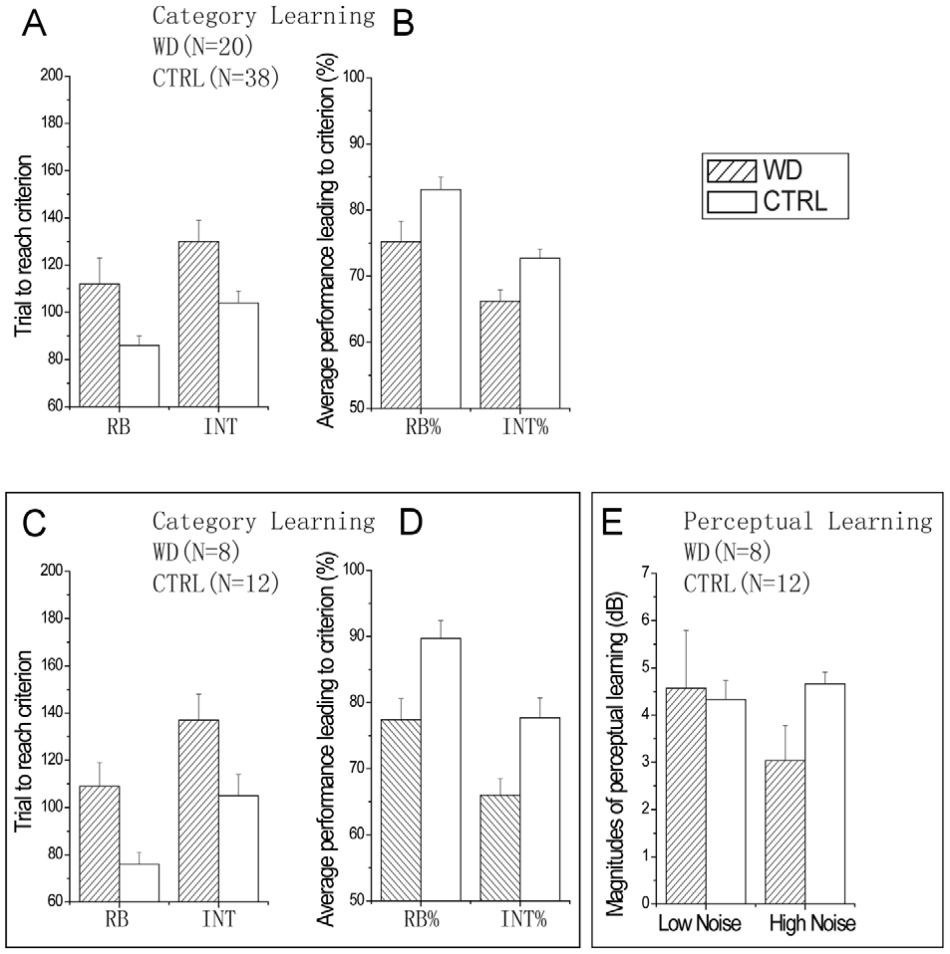
Performance in the category learning tasks. Trial to reach criterion (A) and average performance leading to criterion (B) in rule-based and information-integration category learning for all the WD and normal subjects. (C, D) Trial to reach criterion and average performance leading to criterion in rule-based and information-integration category learning for the WD and normal subjects who completed both category learning and perceptual learning tasks. (E) Magnitudes of perceptual learning in low and high external noise for 8 WD and 12 normal subjects.

It took on average 112±11 and 130±9 trials for the WD group, and 86±4 and 104±5 trials for the normal controls to reach criterion in the rule-based and information-integration category learning tasks, respectively (Figure 4A). Compared to the normal controls, the WD group had significantly slower learning rates in both rule-based and information-integration category learning (t(56) = 2.655 and 2.895, both p<0.01). The average performance level, calculated from all the trials leading to criterion performance, was 75.2±3.1% and 66.2±1.7%, and 83.1±1.9% and 72.7±1.4% for the WD and control groups in the rule-based and information-integration learning tasks (Figure 4B). The WD group also showed significantly worse accuracy than the control group in both category learning tasks (t(56) = 2.286 and 2.809, both p<0.05). The pattern of results held for the one WD patient with visible cortical pathology and the other 19 without it (all p<0.05).

For both the WD and control groups, the two forms of category learning were however not significantly correlated (p>0.10). Following category learning, all the subjects could correctly state the rule they used in performing the rule-based category learning task. In contrast, although some participants could articulate parts of the rule in the information-integration category learning task, none could correctly state it in its entirety.

In summary, the WD subjects exhibited significant deficits in both category learning tasks. Performance in the two category learning tasks was not significantly correlated in each group. Although we can no strong inference can be made from the null result, the pattern is nonetheless consistent with the hypothesis that different nucleus of the basal ganglia may underlie the two forms of category learning [45], [46].

### Perceptual Learning

At an average of 88.0±3.8% correct, the performance of the 8 WD subjects in the central task was statistically equivalent to that of the twelve normal controls at 90.3±1.4% correct (p>0.50).

**Figure 5:**
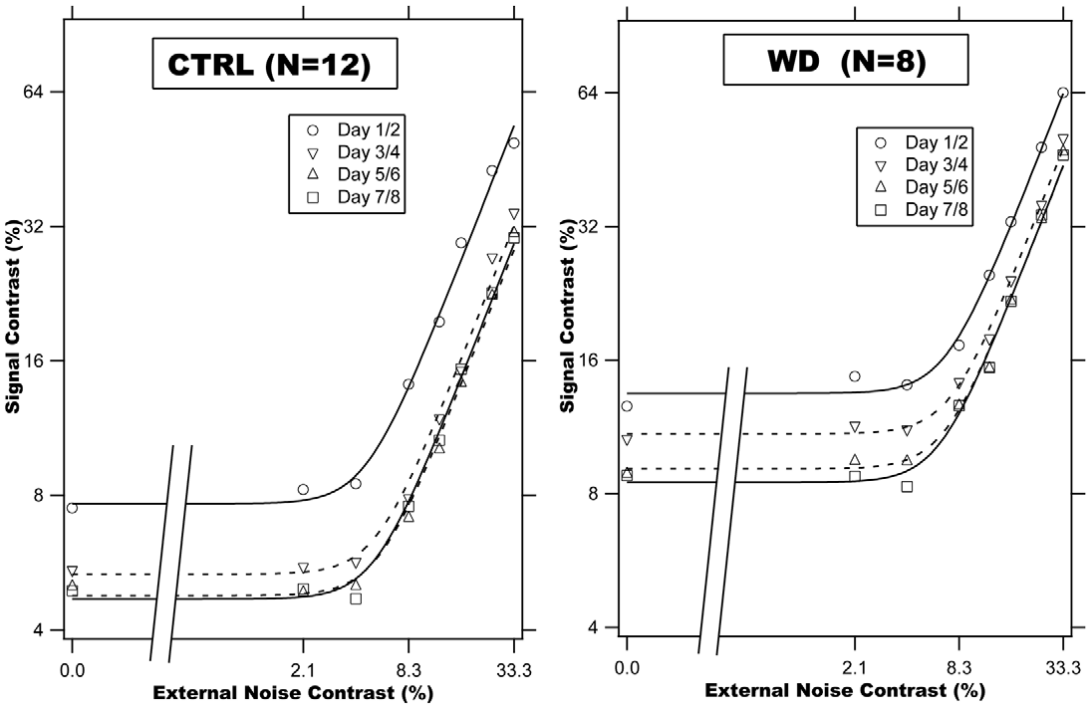
Threshold versus external noise contrast (TvC) functions. Smooth curves represent predictions of the best fitting Perceptual Template Model. Because log(0) is 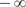, the diagonal lines indicate that a large part of the x-axis is omitted.

Using the staircase procedure, we obtained contrast threshold in the Gabor orientation identification task in each of the eight external noise conditions in every training session. The data are organized in terms of threshold versus external noise contrast (TvC) functions. Figure 5 shows the average threshold estimates of each group a function of external noise level across every two training sessions. The variation in external noise produced curves with the typical structure of TvC functions, flat at low levels of external noise and increasing at higher levels of external noise.

In the lowest three external noise conditions, the average contrast thresholds of day 1/2 are 0.12±0.03 and 0.09±0.01 for the WD and control subjects, respectively, with no significant difference between them (t(18) = 1.371, p>0.10). Perceptual learning reduced the average contrast threshold by 4.57±1.22 dB for the WD subjects, and by 4.33±0.40 dB for the control subjects (Figure 4E). The two groups did not significantly differ in the magnitude of perceptual learning in the low external noise conditions (t(18) = 0.217, p>0.80). The time course of perceptual learning was also not significantly different between the two groups (all p>0.10): The average contrast threshold reduction in every two consecutive training sessions are 2.14±0.52, 1.73±0.68, and 0.71±0.24 in the WD group, and 3.15±0.54, 0.98±0.18, and 0.20±0.26 in the control group.

In the highest two external noise conditions, the average contrast thresholds of day 1/2 are 0.56±0.07 and 0.46±0.07 for the WD and control subjects, respectively, with no significant difference between them (t(18) = 0.194, p>0.30). Perceptual learning reduced the average contrast threshold by 3.04±0.74 dB for the WD group, and 4.66±0.25 dB for the control group. The magnitude of perceptual learning in the high external noise conditions was significantly greater in the normal group than in the WD group (t(18) = 2.425, p<0.03).

### Mechanisms of Perceptual Learning

To identify the mechanisms of perceptual learning, we fit the perceptual template model (PTM) to the TvC functions using a least-square procedure. The perceptual template model was initially introduced in [50] and first applied to perceptual learning in [25], [51]. An in-depth review of the external noise methods and observer models can be found in [52].

**Figure 6:**
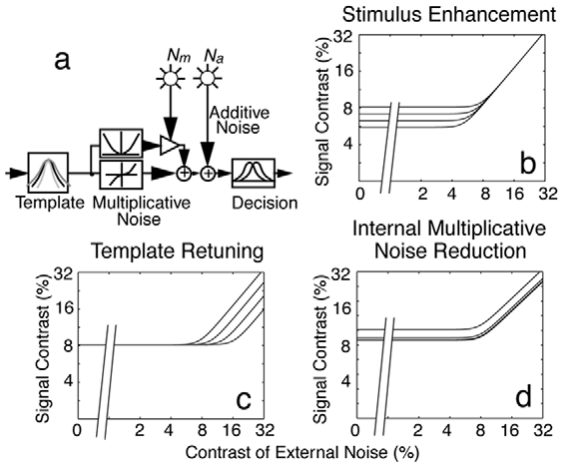
The Perceptual Template Model. (a) The PTM. (b, c, d) Performance signatures of the three mechanisms of perceptual learning.

The PTM consists of five components (Figure 6): (1) a perceptual template (e.g., a spatial frequency filter) with a contrast gain to the signal *β* that is normalized relative to its gain to the external noise, (2) a nonlinear transducer function, which raises its input to the 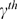 power, (3) a Gaussian-distributed internal multiplicative noise with mean 0 and standard deviation that is proportional to (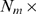) the contrast energy in the input stimulus, (4) a Gaussian-distributed additive internal noise with mean 0 and a “constant” standard deviation 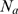, and (5) a decision process. In the PTM, accuracy of perceptual task performance is indexed by 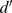 [50]:
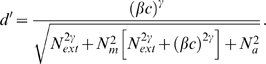
(1)


For a given performance level, *d*′, we can solve Eq. 1 to express threshold contrast *c_τ_* as a function of 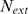 in log form:
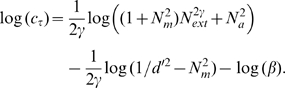
(2)


For an observer described by the PTM, perceptual learning could only improve its performance via one or a combination of three mechanisms: (1) Stimulus enhancement turns up the gain of the perceptual template to the input (both the signal and the external noise), modeled by multiplying 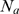 by a factor of 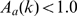 in the learnt condition; (2) External noise exclusion eliminates some of the external noise by tuning the perceptual template around the signal-valued stimulus, modeled by multiplying 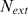 by a factor of 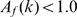 in the learnt condition; and (3) Internal multiplicative noise reduction changes the contrast gain control properties of the perceptual system, modeled by multiplying *N_m_* by a factor 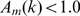 in the learnt condition. If all three mechanisms are operative, the contrast threshold versus external noise function for a PTM becomes:
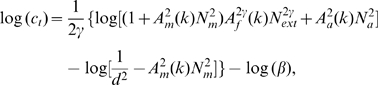
(3)where *c*_t_ denotes the predicted contrast threshold, *N*_ext_ is the standard deviation of external noises, *d′ = 1.634* is the perceptual sensitivity of the observer corresponding to 79.3% correct in the two-alternative forced-choice task, and *k* denotes the learning session.

Although we were fully aware that it is necessary to obtain TvC functions at multiple performance criteria in order to fully constrain the PTM model [53], it was impractical to collect that amount of data with the patients in this study. We have therefore set 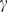 = 1.62 and 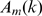 = 1.0, based on the results of [25].

The best fitting PTM model accounted for 95.0±1.0% and 96.0±1.0% of the variance in the WD and normal data, respectively. The parameters of the best fitting model are listed in Table 1. For both groups of subjects, perceptual learning reflects a combination of improved stimulus enhancement and external noise exclusion, with values of 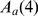 and 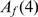 after training of 0.47±0.09 and 0.68±0.06 in the WD group, and 0.47±0.06 and 0.52±0.03 in the normal group. There is no significant difference between 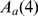
's (t(18) = 0.031, p>0.9) but significant difference between 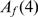
's (t(18) = 2.788, p<0.015) of the two groups. The reduction of 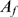 reflects external noise exclusion; it only improves performance in the high external noise conditions. The reduction of 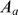 reflects stimulus enhancement via additive internal noise reduction; it only improves performance in the low external noise conditions (Figure 6).

**Table 1:** Best fitting PTM model parameters.

		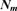	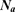	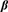	Aa(2)	Aa(3)	Aa(4)	Af(2)	Af(3)	Af(4)	r^2^
WD	CH	0.596	0.014	2.579	0.811	0.575	0.519	0.840	0.652	0.635	0.949
	JW	0.593	0.007	2.743	0.744	0.611	0.561	0.695	0.672	0.655	0.981
	LM	0.594	0.014	2.575	0.649	0.419	0.378	0.762	0.601	0.585	0.982
	XM	0.599	0.003	1.828	0.781	0.798	0.620	0.809	0.653	0.507	0.948
	XX	0.589	0.015	2.630	0.495	0.265	0.243	0.697	0.568	0.548	0.974
	ZG	0.599	0.005	1.614	0.656	0.506	0.411	0.615	0.681	0.705	0.944
	ZH	0.582	0.013	1.860	0.813	0.867	0.914	0.745	0.756	0.820	0.916
	SX	0.606	0.051	1.285	0.423	0.134	0.106	0.766	0.818	0.998	0.886
CTRL	BP	0.607	0.006	1.400	0.305	0.318	0.206	0.416	0.464	0.457	0.970
	LJ	0.604	0.003	1.850	0.900	0.881	0.732	0.751	0.615	0.493	0.950
	FJ	0.586	0.008	2.673	0.592	0.471	0.457	0.553	0.449	0.452	0.980
	RX	0.572	0.010	2.852	0.644	0.524	0.523	0.636	0.544	0.530	0.987
	PX	0.601	0.003	1.624	0.710	0.703	0.598	0.587	0.492	0.497	0.954
	PZ	0.535	0.022	2.939	0.768	0.568	0.589	0.654	0.592	0.578	0.918
	QZ	0.594	0.007	2.715	0.427	0.280	0.256	0.772	0.641	0.545	0.984
	WY	0.589	0.014	2.702	0.438	0.343	0.415	0.808	0.715	0.725	0.962
	NL	0.606	0.007	2.557	0.408	0.326	0.305	0.521	0.451	0.444	0.984
	YY	0.581	0.009	2.742	0.914	0.718	0.842	0.688	0.718	0.686	0.975
	ZB	0.606	0.008	2.561	0.334	0.237	0.280	0.320	0.426	0.421	0.949
	ZY	0.601	0.002	1.213	0.363	0.379	0.388	0.662	0.395	0.402	0.938

### Relationship between Perceptual Learning and Category Learning

For the eight WD and twelve normal subjects who completed both category learning and perceptual learning tasks, a number of additional statistical tests were performed. First, we compared their performance in category learning. We found that the eight WD subjects had much slower learning rates in both rule-based (109±10 versus 76±5 trials, t(18) = 3.354, p<0.01) and information-integration (137±11 versus 105±9 trials, t(18) = 2.591, p<0.02) category learning (Figures 4CD). They also performed with significantly less accuracy in trials leading to criterion (77.4±3.2% vs 89.7±2.7%, t(18) = 2.930, p<0.01 ; 66.0±2.5% vs 77.7±3.0%, t(18) = 2.798, p<0.02).

A stepwise regression analysis was used to evaluate the relationship between the two forms of category learning and perceptual learning in low and high external noise conditions:
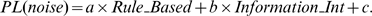
(4)The average amount of perceptual learning in the lowest three external noise conditions and the two highest external noise conditions were used as measures of perceptual learning. In two separate analyses, the trials to reach criterion and the average performance leading to criterion are used as measures of rule-based and information-integration category learning.

**Figure 7:**
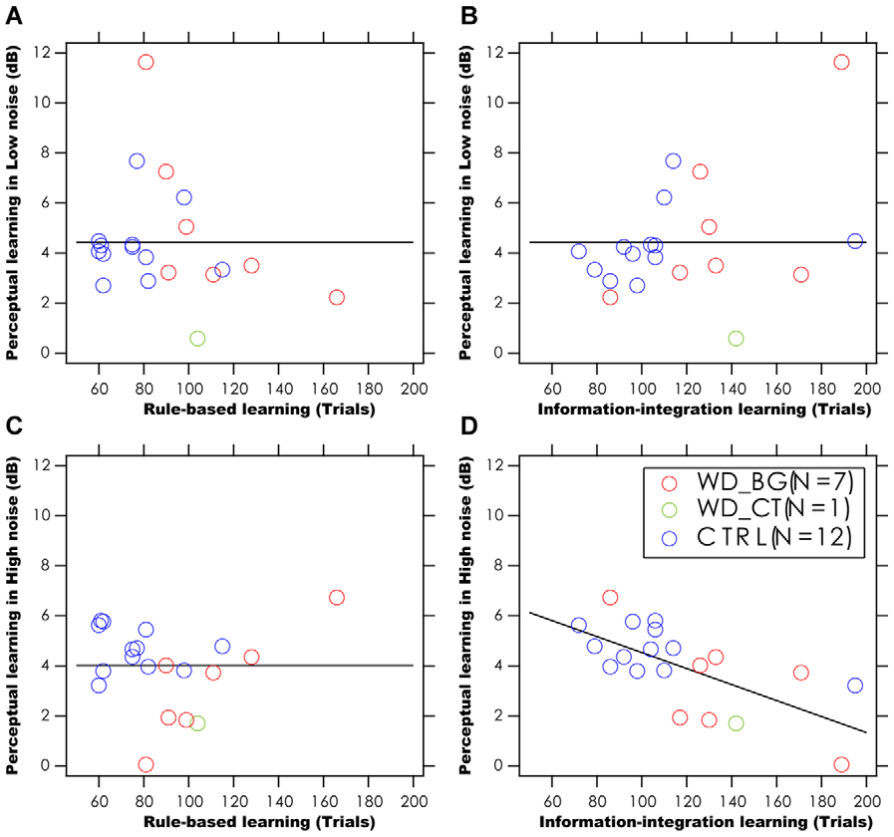
The relationship between perceptual learning and category learning. Scatter plot of the magnitude of perceptual learning versus performance in category learning (trial to reach criterion). The data are shown for 7 WD patients without visible cortical pathology (WD_BG, red), 1 WD patient with visible cortical pathology (WD_CT, green) and 12 normal subjects (CTRL, blue): perceptual learning in low external noise versus rule-based learning (A), versus information-integration learning (B); perceptual learning in high external noise versus rule-based learning (C), versus information-integration learning (D).

**Figure 8:**
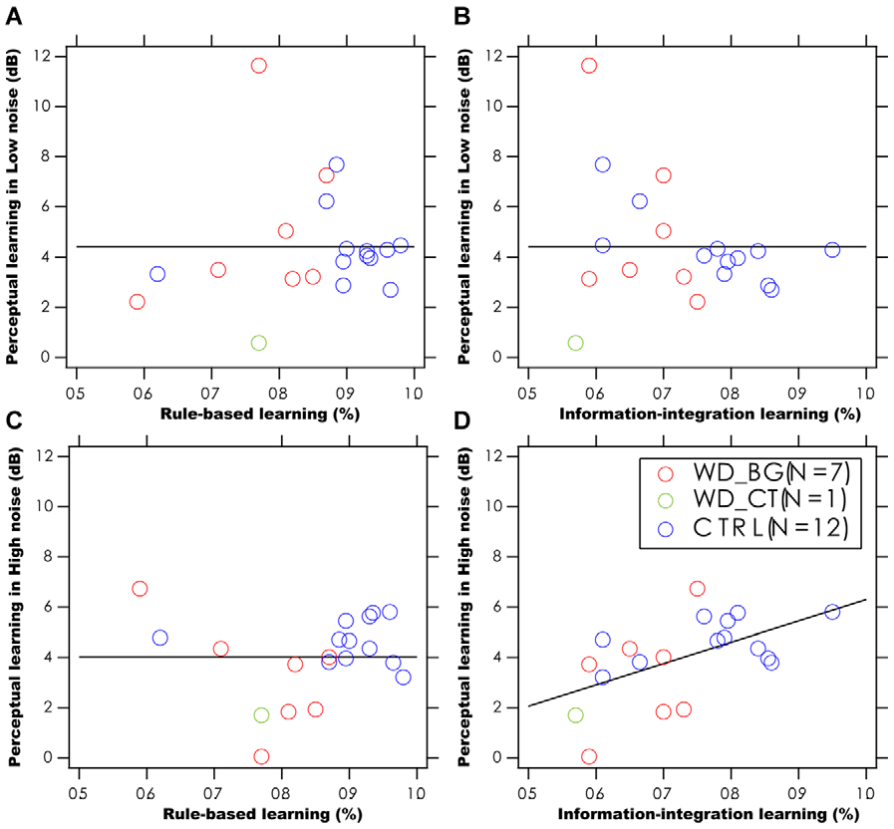
The relationship between perceptual learning and category learning. Scatter plot of the magnitude of perceptual learning versus performance in category learning (average performance accuracy). The data are shown for 7 WD patients without visible cortical pathology (WD_BG, red), 1 WD patient with visible cortical pathology (WD_CT, green) and 12 normal subjects (CTRL, blue): perceptual learning in low external noise versus rule-based learning (A), versus information-integration learning (B); perceptual learning in high external noise versus rule-based learning (C), versus information-integration learning (D).

The coefficients of the best fitting regression models are listed in Table 2. Because of the relative small sample size in both the WD and control groups, we combined data from the two groups in the regression analysis. For perceptual learning in low external noise, the model with a single constant term 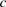 provided statistically equivalent fits to the data in comparison to models with one or both of the other two regressors (p>0.20). This was true when either the number of trials leading to criterion or the average percent correct in both forms of category learning was used as the regressors. On the other hand, for perceptual learning in high external noise, the model with a significant coefficient on information-integration category learning plus a constant, provided statistically equivalent fit to the data in comparison to the full model with three terms (p>0.50), and a superior fit to the data in comparison with the most reduced model with only one constant term (p<0.025). This was true when either the number of trials leading to criterion or the average percent correct in both forms of category learning was used as the regressors. Scatter plots of the magnitude of perceptual learning versus measures of category learning are shown in Figures 7 and 8.

**Table 2:** Correlation Table.

Regressors	Perceptual Learning Condition	a	b	c	P
Trials to reach criterion	PL(low)	0	0	4.42±0.53	1.0
	PL(high)	−0.03±0.01	0	7.73±1.02	0.001
Average Percent Correct	PL(low)	0	0	4.42±0.53	1.0
	PL(high)	8.49±3.01	0	−2.19±2.22	0.011

## Discussion

In this study, we evaluated rule-based and information-integration category learning and perceptual learning in both low and high external noise environments in subjects with treated Wilson's disease and normal controls. The WD subjects exhibited deficits in both forms of category learning as well as perceptual learning in high external noise. However, their perceptual learning in low external noise was relatively spared. There was no significant correlation between the two forms of category learning, nor between perceptual learning in low external noise with either form of category learning. The pattern of results revealed a novel and highly selective relationship between perceptual learning in high external noise and information-integration category learning. Perceptual learning in high external noise was only significantly correlated with information-integration category learning, but not with rule-based category learning.

The observed deficits of WD subjects in both forms of category learning were largely expected, because the primary damage of our WD subjects was in the basal ganglia, a structure that has been known to be important for both forms of category learning [45], [46]. It is interesting that, for both the normal and the WD patients, performance in the rule-based and information-integration category tasks was not significantly correlated. Although no strong inference can be made from the null result, the pattern is nonetheless consistent with the hypothesis that rule-based and information-integration category learning may involve different brain regions [45], [46].

In high external noise environments, perceptual learning improves observer performance by re-tuning the task-relevant perceptual template. Re-tuning the perceptual template is essentially a process of discovering the optimal category structure from the sensory information for the perceptual task, which is highly similar to the category learning process [54]. That WD subjects learned significantly less in high external noise in the perceptual learning task and the significant correlation between perceptual learning in high noise and information-integration category learning suggests that there may be a strong link between information-integration category learning and perceptual learning in high external noise. Damage to brain structures that are important for information-integration category learning may lead to poor perceptual learning in high external noise.

Perceptual learning in low external noise is accomplished by stimulus enhancement, a process that strengthens the internal representation of the input stimuli, independent of the task relevance of the components in the stimuli, and does not involve any categorization structure. That WD subjects did not exhibit significant deficits in perceptual learning in low external noise and the lack of significant correlation between perceptual learning in low external noise and either forms of category learning suggests that the brain structures damaged by the Wilson's disease, mostly the basal ganglia, might not be important for perceptual learning in low external noise.

In a related line of research, it has been shown that subjects with dyslexia exhibit selective deficits in information-integration but not in category learning [49]. They are also selectively impaired in simple visual tasks in high external noise but not in low external noise conditions [55], [56].

There has been mounting evidence from psychophysics on normal observers that stimulus enhancement and template re-turning are two independent mechanisms of perceptual learning [27], [57], [58]. The current study shows that learning in high and low external noise conditions are differentially correlated with category learning and impacted by brain damages in Wilson's disease, supporting the functional distinction between the two mechanisms. L

We speculate that the basal ganglion might be an important brain structure involved in perceptual learning in high external noise conditions. This is an area that has not been explored in perceptual learning research. Future research using single or multi-unit recording, patients, or functional imaging might help us further elucidate the brain structures involved in perceptual learning in human observers.
